# HOUSING AND HOME MODIFICATION FOR AGING IN PLACE: PERSPECTIVES, PROGRESS, AND FUTURE DIRECTIONS

**DOI:** 10.1093/geroni/igad104.1621

**Published:** 2023-12-21

**Authors:** Jon Pynoos

**Affiliations:** University of Southern California, Los Angeles, California, United States

## Abstract

Aging in place remains a high priority of older adults. However, the home environments in which they reside often have hazards and lack supportive features, putting them at risk of falls and making it difficult to carry out daily activities. People who need home modifications the most (i.e., vulnerable populations) are the least likely to have them. In response to the needs of the growing population of older adults, there has been notable progress in the field. Now, with an increasing recognition of the impact of housing on health, there are new opportunities to move the field forward. Highlighting Powell Lawton’s contributions as a foundation, this lecture will: 1) review major developments in research, service delivery, policy, and consumer education, and 2) discuss recommendations for action that practitioners, researchers, policymakers, and advocates could undertake to realize Lawton’s vision of home environments that support aging for all.

